# Targeting fat mass and obesity-associated protein mitigates human colorectal cancer growth *in vitro* and in a murine model

**DOI:** 10.3389/fonc.2023.1087644

**Published:** 2023-02-17

**Authors:** Thuy Phan, Vu H. Nguyen, Rui Su, Yangchan Li, Ying Qing, Hanjun Qin, Hyejin Cho, Lei Jiang, Xiwei Wu, Jianjun Chen, Marwan Fakih, Don J. Diamond, Ajay Goel, Laleh G. Melstrom

**Affiliations:** ^1^ Department of Surgery, City of Hope National Medical Center, Duarte, CA, United States; ^2^ Department of Hematology, City of Hope National Medical Center, Duarte, CA, United States; ^3^ Beckman Research Institute, Department of Systems Biology, City of Hope National Medical Center, Monrovia, CA, United States; ^4^ Beckman Research Institute, The Integrative Genomics Core, City of Hope National Medical Center, Duarte, CA, United States; ^5^ Department of Molecular and Cellular Endocrinology, City of Hope National Medical Center, Duarte, CA, United States; ^6^ Department of Medical Oncology, City of Hope National Medical Center, Duarte, CA, United States; ^7^ Department of Molecular Diagnostics and Experimental Therapeutics, City of Hope National Medical Center, Monrovia, CA, United States

**Keywords:** colorectal cancer, FTO, FTO inhibitor, RNA-seq, signaling pathways

## Abstract

**Introduction:**

Colorectal cancer (CRC) remains a significant cause of cancer related mortality. Fat mass and obesity-associated protein (FTO) is a m6A mRNA demethylase that plays an oncogenic role in various malignancies. In this study we evaluated the role of FTO in CRC tumorigenesis.

**Methods:**

Cell proliferation assays were conducted in 6 CRC cell lines with the FTO inhibitor CS1 (50-3200 nM) (± 5-FU 5-80 mM) and after lentivirus mediated FTO knockdown. Cell cycle and apoptosis assays were conducted in HCT116 cells (24 h and 48 h, 290 nM CS1). Western blot and m6A dot plot assays were performed to assess CS1 inhibition of cell cycle proteins and FTO demethylase activity. Migration and invasion assays of shFTO cells and CS1 treated cells were performed. An in vivo heterotopic model of HCT116 cells treated with CS1 or with FTO knockdown cells was performed. RNA-seq was performed on shFTO cells to assess which molecular and metabolic pathways were impacted. RT-PCR was conducted on select genes down-regulated by FTO knockdown.

**Results:**

We found that the FTO inhibitor, CS1 suppressed CRC cell proliferation in 6 colorectal cancer cell lines and in the 5-Fluorouracil resistant cell line (HCT116-5FUR). CS1 induced cell cycle arrest in the G2/M phase by down regulation of CDC25C and promoted apoptosis of HCT116 cells. CS1 suppressed in vivo tumor growth in the HCT116 heterotopic model (p< 0.05). Lentivirus knockdown of FTO in HCT116 cells (shFTO) mitigated in vivo tumor proliferation and in vitro demethylase activity, cell growth, migration and invasion compared to shScr controls (p< 0.01). RNA-seq of shFTO cells compared to shScr demonstrated down-regulation of pathways related to oxidative phosphorylation, MYC and Akt/ mTOR signaling pathways.

**Discussion:**

Further work exploring the targeted pathways will elucidate precise downstream mechanisms that can potentially translate these findings to clinical trials.

## Introduction

Colorectal cancer (CRC) is the most common gastrointestinal malignancy and the second leading cause of cancer-related death, with an estimated greater than 150,000 diagnosed and 50,000 deaths in the United States in 2020 (1). Treatments for CRC includes conventional therapies such as surgery, chemotherapy, immunotherapy, and radiation. The 5-year survival rate for CRC is 64% at all stages and decreases to only 14% for metastatic disease (2). Interestingly, recent study found out that some specific gut microorganisms playing an important role in resistant to 5-Fluorouracil (5-FU) and oxaliplatin therapy *via* modulating autophagy pathway (3). On the other hand, the use of antibiotics might reduce gut microbiota which is associated with lower mortality in metastatic CRC patients with bevacizumab therapy (4). Therefore, further investigation is needed to develop novel and effective systemic therapies for this disease. N6-methyladenosine (m6A) is the most abundant and prevalent internal modification in eukaryotic mRNA among various types of RNA, including messenger RNA, microRNA, long noncoding RNA and circular RNA (5, 6). m6A modification is the methylation of the adenosine base at the nitrogen-6 position of mRNA and reversibly mediated by the methyltransferase (“writers” including METTL3, METTL14 and WTAP) (7–9), the demethylases (“erasers” including FTO and ALKBH5) (10, 11) and binding proteins that preferentially recognized m6A methylated transcripts and trigger downstream pathways (“readers” including YTH, HNRNP and IGF2BP) (12–14). m6A RNA methylation is also regulated by intestinal bacteria, which is related to the progression of cancers (15, 16). Especially, m6A modifications in CRC cells and patient-derived xenograft markedly suppressed by *Fucobacterium nucleatum via* downregulation of METTL3, which enhances the colorectal metastasis (17). FTO and ALKHB5 belong to human AlkB family of non-heme Fe(II) and 2-oxoglutarate–dependent oxygenases and oxidatively demethylate m6A (10). FTO catalyzes the conversion of m6A to hm6A with slow release of A and formaldehyde (FA), while ALKBH5 directly demethylates m6A to A with rapid FA formation (18). Fat mass and obesity-associated protein (FTO), which plays a role in regulating fat mass and adipogenesis, was identified as the first m6A RNA demethylase (10). Recent studies demonstrated that FTO is overexpressed and plays oncogenic roles in various cancers such as acute myeloid leukemia, gastric cancer, breast cancer, melanoma, and cervical cancer (19–23). Meanwhile, other studies indicated that FTO could function as a tumor suppressor and downregulation of FTO promotes cancer development in renal cell cancer, liver cancer and ovarian cancer (24–27). Importantly, the role of FTO in CRC tumorigenesis through the m6A pathway remains controversial. Recently, Lin et al. demonstrated that FTO was overexpressed in primary and 5-FU-resistant CRC tissues and FTO enhances 5-FU resistance through SIVA1-mediated apoptosis pathway (28). Wang et al. also found that FTO promotes CRC development and increases chemotherapy resistance through G6PD/PARP1 demethylation (29). In contrast, Ruan et al. indicated the low expression of FTO in CRC tissues and better prognosis in patients with higher level of FTO protein (30). Inhibition of FTO promotes cancer stem cell properties in CRC including sphere formation, tumor development and chemoresistance (31). Therefore, understanding the role of FTO in CRC is very important, especially to find out the effective target for treatment to fight against cancer.

In this study, we demonstrated that FTO is expressed in different CRC cell lines. To explore the biological function of FTO in CRC, we conducted knockdown of *FTO* and analyzed changes in proliferation, migration, and invasion of cultured cells *in vitro* and in tumor progression *in vivo*. We also investigated the underlying mechanism of FTO inhibition using RNA-sequencing (RNA-seq). Additionally, as proof of principle, CS1, a small molecule FTO inhibitor was evaluated as a promising candidate for CRC treatment (32). The overall aim of this work was to assess the role of FTO in CRC as it pertains to tumor progression.

## Materials and methods

### Tumor cell line and mice

Human colorectal cancer cell lines (CRC) including *KRAS* wild type (HT-29, COLO) and *KRAS* mutant (HCT116, LoVo, SW480, SW620) were purchased from American Type Culture Collection (ATCC, USA). 5-FU resistant HCT116 cell line was obtained from MD Anderson Cancer Center (USA). Cells were cultured in McCoy for HT-29 or DMEM for COLO, HCT116, HCT116-5FUR, SW480, SW620 or F-12K for LoVo (all media from Corning, USA). Media were supplemented with 10% FBS (Gibco, USA) and 1% penicillin-streptomycin (Gibco, USA) and cells were grown at 37°C in a humidified incubator with 5% CO_2_.

6-week-old female NSG mice were purchased from Jackson Lab and maintained under specific pathogen-free conditions at Animal Research Center (City of Hope). Animals were handled according to Institutional Animal Care and Use Committee (IACUC) guidelines under an approved protocol #16067.

### Reagents

FTO inhibitor, CS1 or bisantrene (B4563, Sigma-Aldrich) was dissolved in DMSO and saturated β-cyclodextrin (C0926, Sigma-Aldrich) solution for *in vitro* and *in vivo* experiments, respectively.

### Cell proliferation assay

The proliferation of CRC cell lines under CS1 treatment was determined using the MTS assays (Cell Titer 96 Aqueous One Solution, Promega, USA). Cells (1x10^4^ cells/100 µl/well) were seeded in 96-well tissue culture plates. After 24 h, cells were incubated with CS1 (50-3200 nM) as single agent. In combination treatment, HCT116 cells were incubated with CS1 and 5-FU (5-80 µM). After 72 h of treatment, cell proliferation was evaluated using an MTS assay according to the manufacturer**’**s instructions. Briefly, 20 μl of MTS reagent was added to each well, and the plates were incubated for 2 h at 37°C. Absorbance at 492 nm was measured with a microplate reader (Filtermax F3). Results were represented as the means ± standard deviation of the mean (SD) from triplicate wells.

### Cell cycle analysis

HCT116 cells were seeded into 6-well plates (5x10^5^ cells/well), treated with 290 nM CS1 or DMSO (vehicle control) for 24 h and 48 h. Then cells were harvested, fixed in 70% ice-cold ethanol for >1 h at 4°C, washed twice with cold PBS, and treated with 100 µg/mL ribonuclease, before staining with 50 µg/mL propidium iodide. Subsequently, the stained cells were measured by LSR Fortessa flow cytometer (BD, USA) and analyzed by Flowjo software.

### Apoptosis assay

FITC Annexin V and PI double staining was used to determine apoptosis (Apoptosis Detection Kit I, BD, USA). HCT116 cells were seeded into 6-well plates (5x10^5^ cells/well), treated with 290 nM CS1 or DMSO (vehicle control) for 24 h and 48 h. According to the manufacturer’s protocol, the cells were harvested, washed with Annexin V binding buffer, and stained with PI and Annexin solution for 15 min at room temperature in the dark. The stained cells were measured by LSR Fortessa flow cytometer (BD, USA) and analyzed by Flowjo software.

### *FTO* knockdown in HCT116 cell line using Lentivirus

For lentivirus production, HEK293T cells were co-transfected with Lentiviral vector pLKO.1-shScr (Scramble control), pLKO.1-shFTO-A (Sigma) targeting *FTO* gene and packaging plasmids (TR30037, Origene) using TurboFectin transfection reagent (Origene). 24 h after transfection, media was changed and after another 48 h, cell supernatants were harvested, centrifuged, and filtered to collect lentiviral particles. Next, HCT116 cells were transduced with packaged lentiviral particles in the presence of polybrene (8 µg/ml) using the spinoculation method at 1000 g for 1 h. For stable cell line generation, transduced cells were selected with 2 μg/ml puromycin beginning 48 h after infection and maintained under selection for 2-3 passages.

### Western blot analysis

CRC cells were collected and lysed in protein extraction buffer (150 mM NaCl, 10 mM Tris, 1 mM EDTA, 1% NP-40, 1 mM EGTA, and 50 mM NaF) containing protease inhibitor cocktail (Roche) on ice for 30 min, followed by centrifuge at 13,000 rpm at 4°C for 15 min. The supernatants were then collected, and the protein concentration was quantified by the BCA method. The cell lysates were subjected to SDS-PAGE, and subsequently transferred to PVDF membrane (Invitrogen). The membranes were probed with rabbit anti-human FTO (ab124892, Abcam; 1:1000 dilution) or rabbit anti-human β-actin (4970, Cell Signaling; 1:1000 dilution) antibodies, followed by goat anti-rabbit IgG (whole molecule) peroxidase conjugate (A6154, Sigma; 1:2000 dilution). Bioluminescence was catalyzed using a Quick Spray Chemiluminescent HRP Antibody Detection Reagent (Thomas Scientific, E2400), and bands were detected in a luminescent image analyzer PXi (Syngene).

### m6A dot blot assay

HCT116 cells were treated with DMSO or CS1 at 290nM (IC50) for 48 h. Total RNA was extracted using RNeasy Mini Kit (Qiagen), and poly (A)+ RNA was further enriched with PolyATract mRNA isolation System IV (Promega) according to the manufacturer’s instructions. The RNA samples were diluted in RNA binding buffer, and denatured at 65°C for 5 min. Then one volume of 20X SSC buffer was added into the RNA samples before dotted onto the Amersham Hybond-N+ membrane (GE Healthcare) with Bio-Dot Apparatus (Bio-Rad). The RNA samples were cross-linked onto the membrane *via* UV irradiation for 5 min. The membrane was stained with 0.02% methylene blue (MB) as loading control. After that, the membrane was washed with 1X PBS-T buffer, blocked with 5% nonfat dry milk and incubated with rabbit anti-m6A antibody (202003, Synaptic Systems, 1:2000 dilution) at 4°C overnight. Finally, the membrane was incubated with the HRP-conjugated goat anti-rabbit IgG (sc-2030, Santa Cruz Biotechnology) and developed with Amersham ECL Prime Western Blotting Detection Reagent (GE Healthcare).

### Tumor challenge and therapy

Stably transduced HCT116 shScr or HCT116 shFTO-A cell lines (5 × 10^5^) were suspended in PBS and injected subcutaneously into the right thigh of NSG mice (n=3/group). Two weeks after implantation, the tumors were measured once a week by a caliper and tumor volume (mm^3^) was calculated using the formula 1/2 x Length x Width x Depth.

For studying the therapeutic effect of CS1, HCT116 cells (5 × 10^5^) were suspended in PBS implanted subcutaneously into the right thigh of NSG mice (n=4-5/group). Three weeks after tumor inoculation, when the tumor sizes reached 50 mm^3^, the mice were randomized into 2 groups: control and CS1. Mice were administrated with total of 15 doses of CS1 (5 mg/kg) or control vehicle (β-cyclodextrin) every other day by intraperitoneal injection. Tumor volume (mm^3^) was measured once a week with a caliper until the tumor volume exceeded 1000 mm^3^ or any experimental endpoint, as pre-determined in the IACUC protocol.

### Cell migration and invasion

For migration assays, 5 × 10^4^ cells (HCT116 shScr and HCT116 shFTO-A) were plated in the top chamber of the non-coated insert (Corning, USA) with 8 µm pore size. For invasion assays, 1 × 10^5^ cells were plated in the top chamber of Matrigel-coated insert with 8 µm pore size. Cells were seeded in 200 μl low-serum (1% FBS) DMEM (upper chamber) and placed in 24-well plates (lower chamber) containing 800 μl of high-serum (10% FBS) DMEM. After 24 h of incubation at 37°C, the cells remaining in the upper side of the insert membrane were gently scraped off with cotton swabs. The cells that migrated or invaded through the pores to the lower surface of the insert membrane were fixed in 4% formaldehyde, stained with 1% crystal violet dye, and then counted under a microscope. The values were calculated by averaging the total number of cells from different microscopic fields of three chambers per condition.

### RNA sequencing and data analysis

Total RNA samples were isolated from HCT116 shScr or HCT116 shFTO-A cells with RNeasy Mini Kit (Qiagen, USA) for sequencing. RNA concentration was measured by NanoDrop 1000 (Thermo Fisher Scientific, USA) and RNA integrity was determined using Bioanalyzer (Agilent). Each RNA sample was spiked in with an appropriate amount of either Mix1 or Mix2 according to Life Technologies’ guidelines which would lead to about 1% of the total number of RNA-Seq reads mapping to the 92 ERCC control sequences, assuming the mRNA fraction in the total RNA is 2%. Library construction of 300 ng total RNA for each sample was made using KAPA Stranded mRNA-Seq Kit (Illumina Platforms) (Kapa Biosystems, Wilmington, USA) with 10 cycles of PCR amplification. Libraries were purified using AxyPrep Mag PCR Clean-up kit (Thermo Fisher Scientific). Each library was quantified using a Qubit fluorometer (Life Technologies) and the size distribution assessed using the 2100 Bioanalyzer (Agilent Technologies, Santa Clara, USA). Sequencing was performed on an Illumina Hiseq 2500 (Illumina, San Diego, CA, USA) instrument using the TruSeq SR Cluster Kit V4-cBot-HS (Illumina) to generate 51 bp single-end reads sequencing with v4 chemistry. Quality control of RNA-Seq reads was performed using FastQC. Each group contains 3-4 replicates. Reads were trimmed for adaptor sequence, masked for low complexity or low-quality sequence by Cutadapt (33), and then aligned to reference genome GRCh38 by STAR (34). The expressions of the genes were calculated using RSEM (35), p < 0.05 was set as the threshold of the differential expressions. The reads distributed in a specific transcript were displayed by IGV (36). Hierarchical cluster analysis was generated by R package cluster. Gene Set Enrichment Analysis (GSEA) and hallmark gene sets in Molecular Signatures Database (MSigDB) (37) were applied for enriched pathways.

### Quantitative real-time PCR

To study the gene-downregulation related to *FTO*, HCT116 cells were treated with 290nM CS1 or DMSO as control for 48 h. Then, cells (control, CS1-treated HCT116, HCT116-shScr, and HCT116-shFTO-A) were collected and total RNAs were extracted using RNeasy Mini Kit (Qiagen, USA), according to manufacturers’ instructions. The cDNA was synthesized from RNA using RevertAid Reverse Transcriptase (Thermo Fisher Scientific, USA). Next, cDNA was amplified using the RT-PCR primer sets listed in the **Table 1**. PCRs were performed in QuantStudio 3 machine (Thermo Fisher Scientific, USA). Select Master Mix (Applied Biosystem, USA) was used to detect amplification under the following conditions: 2 m at 50°C, 2 m at 95°C followed by 40 cycles of 15 s at 95°C, and 60 s at 60°C. Results were analyzed with QuantStudio Analysis Software. *HPRT* was used as housekeeping gene to assess target gene.

**Table 1:** Primer set for RT-PCR in this study.

*Gene name*	*Primer sequence (5’ → 3’)*
*GAPDH*	F: GCA CCG TCA AGG CTG AGAAC
	R: ATG GTG GTG AAG ACG CCAGT
*EREG*	F: GTG ATT CCA TCA TGT ATC CCA GGAG
	R: AGA TGC ACT GTC CAT GCA AACAA
*KRAP*	F: CAT ATG ACA GAG GAG GAC A
	R: GTG GCT GTC CTG CTT AGG
*PDE4B*	F: ATC TCA CGC TTT GGA GTC AAC
	R: TTA AGA CCC CAT TTG TTC AGG
*KRAP*	F: GAC GTG ATG AAC CAG ATA TTG CT
	R: TTG ACG AAA ACG GCT TGT TAA AG

## Results

### The FTO inhibitor, CS1 inhibits cell proliferation of different human colorectal cancer cell lines *in vitro*

**Figure 1:**
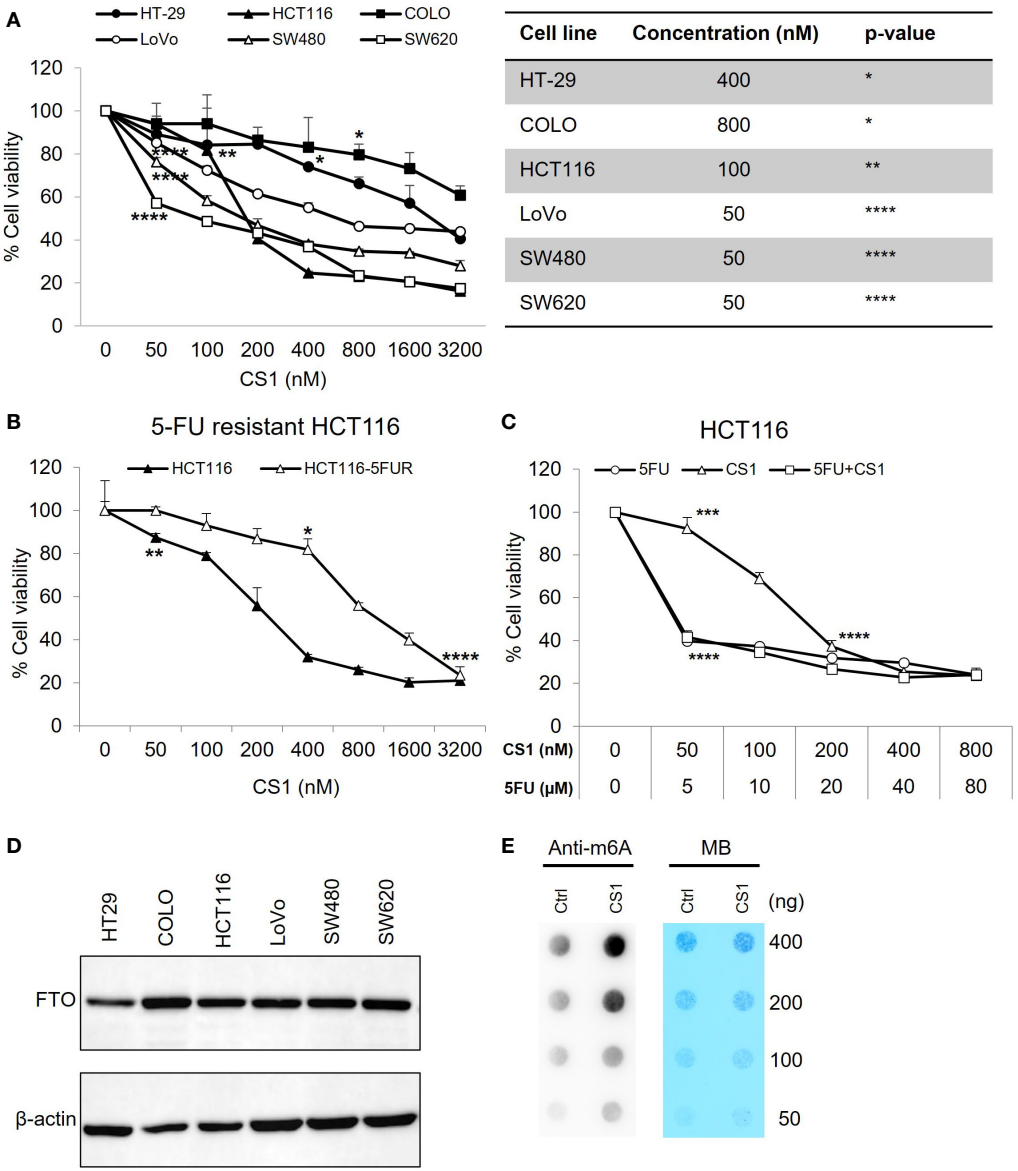
Inhibitory effect of CS1 on different human colorectal cancer cell lines (CRC). **(A)** KRAS wild type (HT-29, COLO) and KRAS mutant (HCT116, LoVo, SW480, SW620) cell lines were seeded in 96 well-plates at a concentration of 1x10^4^ cells/well. 24 h later, cells were treated with CS1 at increasing concentrations (0, 50, 100, 200, 400, 800, 1600 and 3200 nM). 72 h after treatment, MTS assay was applied to evaluate the percentage of cell viability. **(B)** Similar procedure was applied in the parental HCT116 cells and 5-FU resistant derivatives. HCT116 **(C)** were treated with CS1 (0, 50, 100, 200, 400, and 800 nM) and/or 5-FU (0, 5, 10, 20, 40, and 80 µM) as single agents or in combination. Results from one representative experiment are presented as means ± SD, with triplicate determinations. (*) p < 0.05; (**) p < 0.01; (***) p < 0.001; and (****) p < 0.0001. p-value vs control (untreated group) in each cell line. **(D)** CRC cells were collected, lysed, and total protein was obtained. Western blot analysis was performed to examine the expression of FTO. β-actin was used as a loading control. **(E)** Determination of m6A abundance in mRNA in HCT116 cells after 72 h of CS1 treatment *via* dot blot assay. MB (methylene blue) represents loading control of RNA samples.

The cytotoxic effects of the small molecule FTO inhibitor, CS1 was examined in different CRC cell lines (HT-29, COLO, HCT-116, LoVo, SW480, SW620) using an MTS assay. As shown in **Figure 1A**, CS1 (50-3200nM) suppresses the proliferation of CRC cells in a dose-dependent manner after 72 h of treatment (p<0.05). The most significant suppression was observed in HCT116 and SW620 cells (16.23% and 17.37% at 3200 nM respectively). 5-FU-based chemotherapy is the standard approach for colon cancer treatment, and resistance to 5-FU is a major cause of therapeutic failure. Next, we explored if CS1 could inhibit cell growth in a 5-FU resistant HCT116 cell line (HCT116-5FUR). The MTS results indicated that CS1 also suppressed cell viability in a dose-dependent manner (50-3200nM) in HCT116-5FUR (**Figure 1B**) (p<0.05 from 400nM). At a concentration of 3.2µM CS1, the proportion of cell toxicity was comparable in 5-FU resistant HCT116 and parental HCT116 cells. We then explored if the combination of 5-FU and CS1 treatment could enhance the inhibitory effect of each drug alone. HCT116 cells were treated with CS1 (50-800nM) and 5-FU (5-80µM) alone or in combination for 72 h and MTS assay was performed for cell viability. The results showed the similar percentages of cell viability at CS1 200-800nM and 5-FU 20-80µM as single agents and in combination (37.17% to 23.56%), with no findings of synergy (combination index >1 by CompuSyn program, data not shown) between these 2 drugs (**Figure 1C**). The protein expression of FTO in all CRC cells in this study were confirmed by Western blot (**Figure 1D**). To assess the inhibition of FTO by CS1 treatment on mRNA methylation, m6A dot plot assay was performed after HCT116 cells were treated by CS1 (290nM, IC50). The results showed a substantial increase of m6A abundance in transcriptomes of CS1-treated samples compared to the controls (**Figure 1E**). These data indicated that CS1 inhibited the demethylase activity of FTO protein in CS1-treated cells.

### CS1 induces cell cycle arrest in G2/M phase and promotes apoptosis

**Figure 2:**
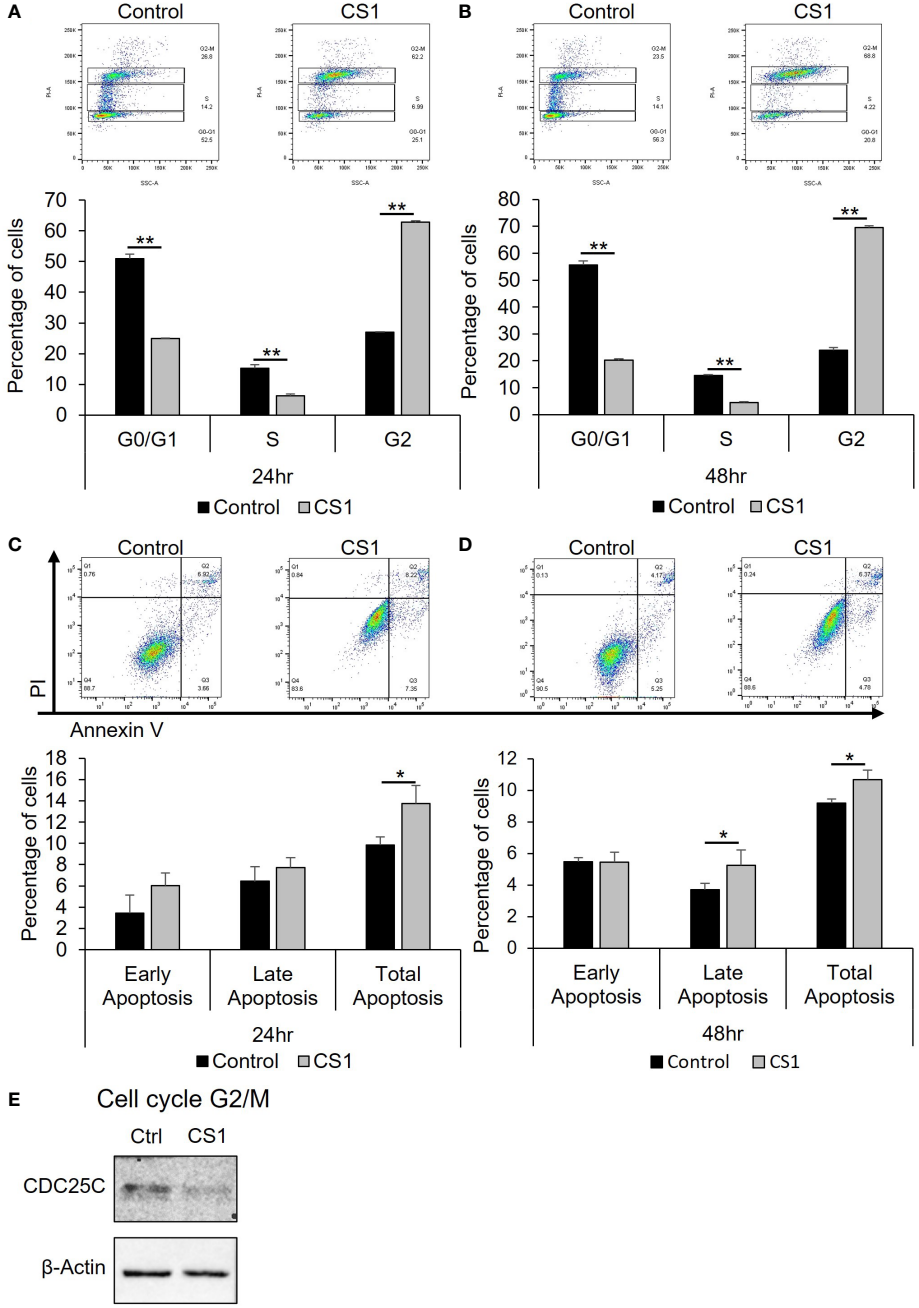
Effect of CS1 in cell cycle and apoptosis. HCT116 cells were seeded into 6-well plates (5x10^5^ cells/well), treated with 290nM CS1 or DMSO (vehicle control) for 24 h and 48 (h) **(A, B)** For cell cycle analysis, cells were fixed in 70% ice-cold ethanol, treated with 100 µg/mL ribonuclease, stained with 50 µg/mL propidium iodide (PI) and then analyzed using flow cytometry. The percentage of cells in G0/G1, S and G2 phases of cell cycle was calculated and displayed. **(C, D)** For cell apoptosis assay, cells were double-stained with Annexin V-FITC+PI and assessed by flow cytometry. The percentages of early apoptotic cells (annexin V (+)/PI (−)), late apoptotic cells (annexin V (+)/PI (+)) and total apoptotic cells were calculated and displayed. **(E)** Western blot analysis of proteins related to G2/M phase (CDC25C) were performed after 48 h of CS1 treatment. β-actin was used as a protein loading control. Data are presented as mean ± SD. of three independent experiments. (*) p <0.05 and (**) p<0.0001..

To investigate how CS1 influences the cell cycle, HCT116 cells were treated with 290nM CS1 or DMSO and then the cell cycle was analyzed using flow cytometry. As shown in **Figures 2A, B**, CS1 treatment resulted in a greater percentage of cells in G2/M phase compared to controls at 24 h (62.8% vs 27.0%) and 48 h (69.6% vs 23.9%) (p<0.0001). CDC25C is one of the crucial regulators that dephosphorylates CDC2 and leads to the activation of the CDK1 complex at the G2/M checkpoint (38). Western blot data indicated that CS1-treated cells demonstrated decreased expression of CDC25C (**Figure 2E**). These data suggest that CS1 may induces G2/M cell cycle arrest in HCT116 cells through downregulation of CDC25C leading to cell growth inhibition. Next, to investigate whether apoptosis contributed to the growth inhibition by CS1, we performed an apoptosis assay using FITC annexin V and propidium iodide double staining in HCT116 cells. There was a significantly higher proportion of total apoptotic cells observed in the CS1-treated groups vs the controls at 24 h (13.8% vs 9.9%) and 48 h (10.7% vs 9.2%) (p<0.05) (**Figures 2C, D**). Overall, these data demonstrate that inhibition of cell growth in CS1-treated HCT116 cells is associated with G2/M cell cycle arrest, decreased CDC25C expression, and the induction of cell apoptosis.

### FTO inhibition suppresses tumor growth in the HCT116 xenograft mouse model

**Figure 3:**
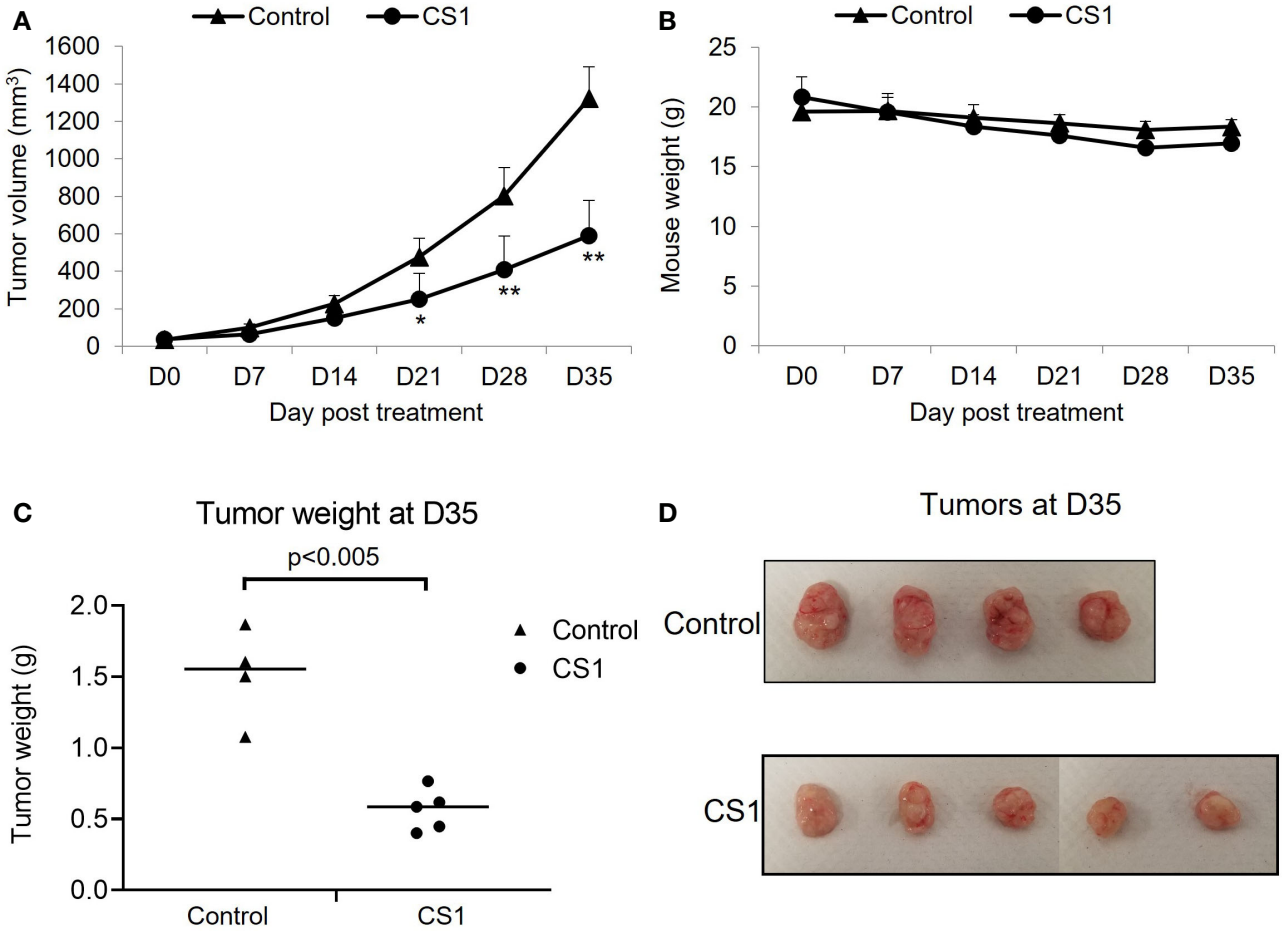
Therapeutic effect of CS1 treatment on HCT116 xenograft mouse model. 6-week-old female NSG mice (n = 4-5/group) were subcutaneously injected into the right thigh with HCT116 cells (5 × 10^5^ cells/mouse). After 21 days, when the tumor volume reached around 50 mm^3^, the mice were randomized into 2 groups: control and CS1. Mice were administrated with total of 15 doses of CS1 (5 mg/kg) or control vehicle (β-cyclodextrin) every other day by intraperitoneal injection. Tumor volume was measured every 7 days until the end of experiment. Mice were then euthanized when the tumor volume reached 1000mm^3^. **(A)** Tumor growth curve. **(B)** Mouse weights. **(C)** Tumor weights and **(D)** the photographs of excised tumors at day 35 after the first treatment. Data are presented as means ± SD. (*) p <0.05 and (**) p<0.0001.

To investigate the *in vivo* antitumor efficacy of the FTO inhibitor, CS1, a xenograft mouse model with HCT116 cells was used. After appropriate growth of tumors, the mice were randomized into 2 groups: control and CS1. As shown in **Figure 3A**, CS1 significantly inhibited tumor progression compared to vehicle, especially from day 21 post-treatment (p<0.05). We also observed no significant differences in mice body weight between the CS1-treated group and controls, which suggested minimal toxicity of CS1 *in vivo* (**Figure 3B**). Consistent with the tumor growth curve, all the tumor masses from HCT116 tumor bearing mice treated with CS1 were found to be substantially smaller than the tumor masses from the control (**Figures 3C, D**).

### Knockdown of *FTO* by employment of Lentivirus-mediated shRNA in HCT116 cells

**Figure 4:**
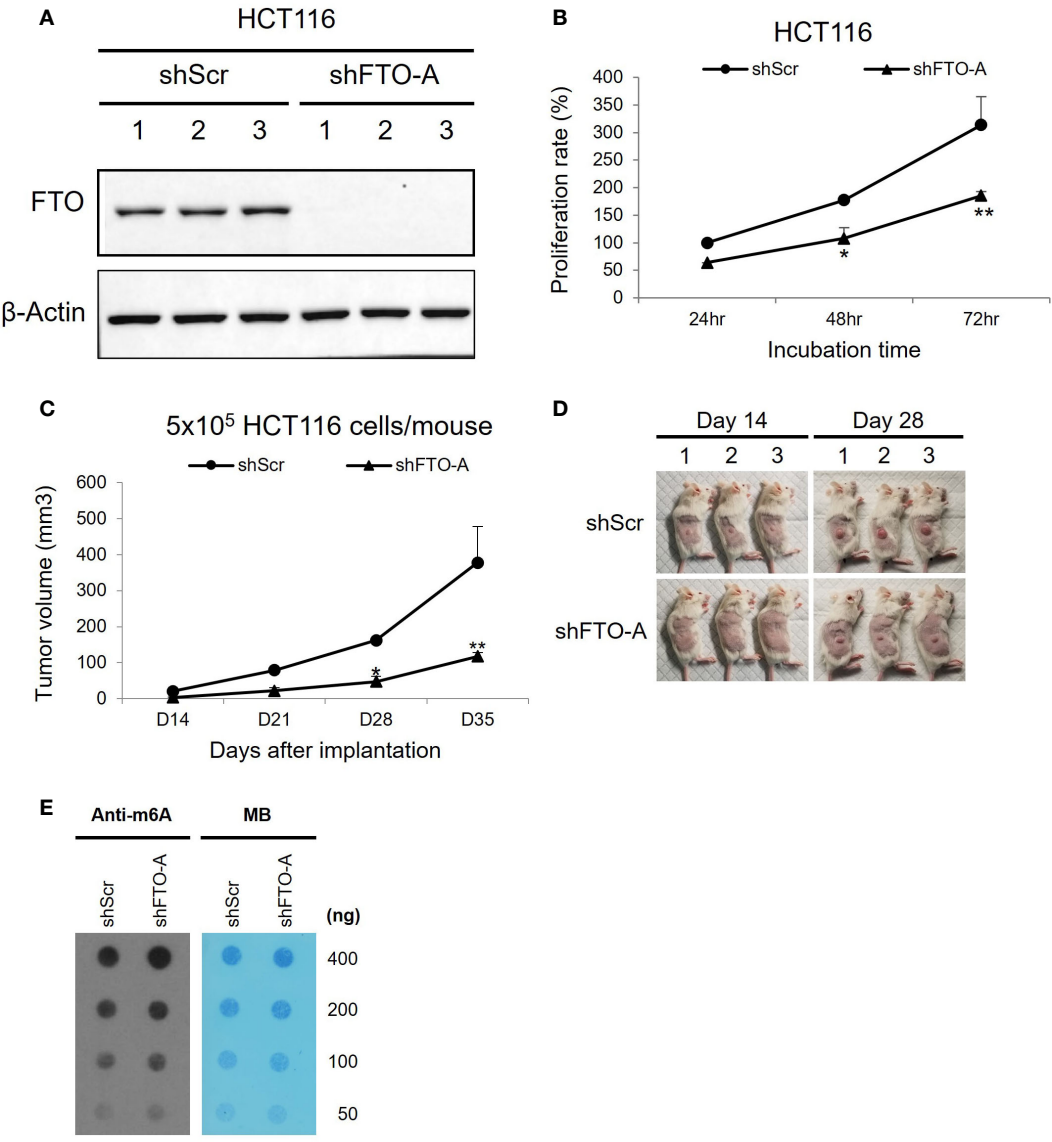
Knockdown of *FTO* in HCT116 cell line using Lentivirus-mediated shRNA. HCT116 cells were transduced with shFTO-A (knockdown group) or shScr (negative control group) lentivirus. Three stably transduced cell lines of each clone were selected using 2 μg/ml puromycin. **(A)** For knockdown efficiency of *FTO* by shRNA lentivirus, Western blotting was performed to detect the FTO expression levels in *FTO* KD (shFTO-A) and negative control (shScr) cells. β-actin was used as a loading control. **(B)** Cell proliferations were examined using MTS assay at 24 h, 48 h and 72 (h) **(C, D)** The effect of *FTO* KD on tumor growth *in vivo* was confirmed by subcutaneous mouse model. 6-week-old female NSG mice (n = 3/group) were injected into the right thigh with HCT116 shScr or HCT116 shFTO-A cell lines (5 × 10^5^). Two weeks after implantation, the tumors were measured once a week by a caliper and tumor volume (mm^3^) was calculated using the formula 1/2 x Length x Width x Depth for tumor growth curve **(C)**. Pictures of tumor bearing mice were taken at day 14 and 28 after tumor inoculation **(D)**. Data are presented as means ± SD. (*) p <0.01 and (**) p<0.0001. **(E)** Global m6A abundance of poly(A)+ RNA isolated from HCT116 shScr or HCT116 shFTO-A cell lines and detected by m6A dot plot assay (left panel). The membrane was stained with 0.02% methylene blue (MB) as loading control (right panel).

To study the role of FTO in colorectal cancer, we used Lentivirus-mediated shRNA targeting *FTO* to inhibit FTO expression. HCT116 cells were stably transduced to express an shRNA sequence (shFTO-A) that specifically silences *FTO*. The *FTO*-knockdown effects were confirmed through Western blot for protein expression. As shown in **Figure 4A**, shFTO-A transduced cell lines have lower expression of FTO protein compared to shScr transduced cells. Additionally, knockdown of *FTO* by shFTO-A markedly inhibited cell growth in HCT116 cells *in vitro* (**Figure 4B**, p< 0.0001). To further investigate the oncogenic role of FTO in colon cancer, we utilized a subcutaneous mouse model. Stable *FTO* knockdown (*FTO* KD) shFTO-A and control shScr transduced in HCT116 cells were injected into the right thigh of the mice. The tumor growth curve of the shFTO-A group was markedly less than control (**Figures 4C, D**). Furthermore, we assessed the effect of *FTO* KD on m6A mRNA levels. To assess the functional impact of *FTO* KD on FTO demethylase activity, we performed a m6A dot plot assay. Knockdown of *FTO* increased m6A mRNA levels, demonstrating inhibition of FTO demethylase activity (**Figure 4E**). Altogether, these findings suggested that FTO has an important role in cell proliferation, tumor progression and m6A demethylation in the HCT116 colon cancer cell line.

### Knockdown of *FTO* inhibited *in vitro* migration and invasion of HCT116 cells

**Figure 5:**
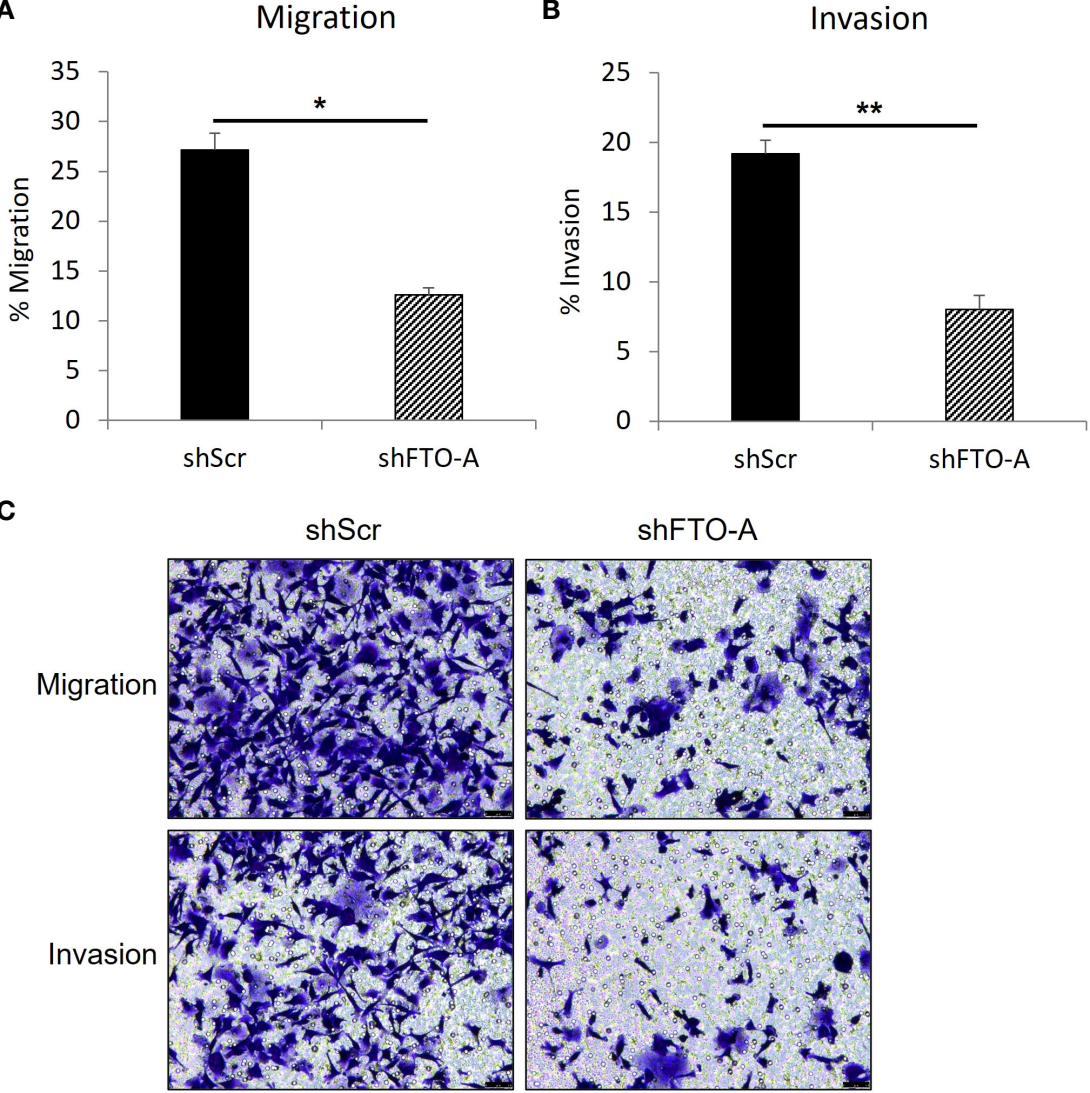
*FTO* knockdown inhibited the migration and invasion of HCT116 cells. Cell migration or invasion was assessed with a transwell assay. Stable *FTO* knockdown (shFTO-A) and negative control (shScr) of HCT116 cell lines were seeded in 1% FBS containing medium in non-coated inserts or in Matrigel-coated inserts (Corning, USA), and were placed in 24 well plates with 10% FBS containing medium. After 24 h, the cells that migrated or invaded through the pores to the lower surface of the insert membrane were fixed, stained, and counted under a microscope. **(A)** Percentage of migrated cells are shown. **(B)** Percentage of invaded cells are shown. **(C)** Representative images are shown. Data are presented as means ± SD from 3 fields. Scale = 50µm. (*) p <0.05 and (**) p<0.0001.

To evaluate the impact of *FTO* KD on cell migration and invasion, the transwell assay was performed on three stably transduced HCT116 cell lines with shFTO-A or negative control shScr. As shown in **Figures 5A, C** (upper panel), after 24hr of incubation, *FTO* KD cells by shFTO-A had a markedly lower percentage of migration compared to the negative controls (p<0.05). Similar to the migration assay, shFTO-A demonstrated significantly less invasion compared to controls (p<0.01) (**Figures 5B, C**, lower panel). These results suggest that shRNA-mediated inhibition of FTO expression decreases the capacity of migration and invasion of HCT116 cells.

### Proposed signaling pathways related to *FTO* knockdown

**Figure 6:**
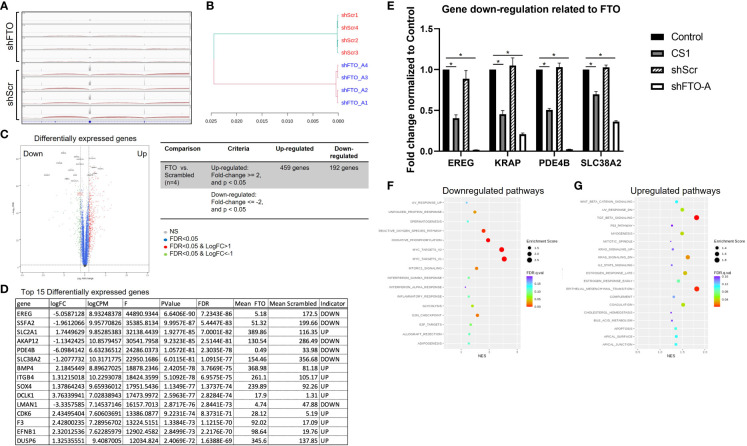
Proposed signaling pathways related to *FTO* knockdown. RNA-seq was performed on mRNA of shScr and shFTO-A from HCT116 cells. **(A)** Distribution of RNA‐seq reads around the genomic locus of FTO in shScr and shFTO using Integrative Genomics Viewer (IGV). **(B)** Hierarchical clustering dendrogram of RNA-seq data from controls and *FTO* KD. **(C)** Volcano plot represents differentially expressed genes in *FTO* KD HCT116 cells compared to shScr control. The red dots and green dots indicate significantly upregulated and downregulated genes, respectively. The blue dots indicate insignificant differentially expressed genes. False Discovery Rate (FDR) is an adjusted p-value for multiple tests (by the Benjamini-Hochberg procedure) by giving the proportion of tests above threshold that will be false positives. FC=fold change. **(D)** Top 15 differentially expressed genes using Ingenuity Pathway Analysis (Qiagen). **(E)** Top 4 downregulated genes from control, CS1-treated, shScr and shFTO HCT116 cells by RT-PCR. Data are presented as means ± SD from 3 fields. (*) p<0.0001. **(F, G)** Scattergrams of the downregulated pathways **(F)** and upregulated pathways **(G)** based on GSEA.

RNA sequencing (RNA-seq) was performed to understand the underlying mechanisms related to *FTO* KD. Distribution of RNA-seq reads around the genomic locus of *FTO* indicating a successful knockdown of *FTO* by shFTO (**Figure 6A**). Next, the hierarchical clustering dendrogram demonstrates that shFTO-A can be grouped together and these separate from the shScr controls (**Figure 6B**), which indicates the consistency and variance of the samples (n=4/group). As compared to HCT116-shScr cells, HCT116-shFTO cells had 459 up-regulated genes and 192 down-regulated genes (**Figure 6C**). Top 15 differentially expressed genes have been shown in **Figure 6D** and top 4 down-regulated genes (*EREG*, *KRAP*, *PDE4B* and *SLC38A2*) were confirmed by qPCR (**Figure 6E**). There were no significant differences between untreated and shScr cells. Similar to *FTO* KD by shFTO, CS1 treatment markedly suppressed these 4 genes compared to controls (**Figure 6E**). A global gene set enrichment analysis (GSEA) revealed a set of downregulated or upregulated pathways in *FTO* KD compared to shScr (**Figures 6F, G**). There were various signaling pathways downregulated by *FTO* KD, including MYC target V1, MYC target V2, oxidative phosphorylation, reactive oxygen species, G2M checkpoint, mTORC1, and unfolded protein response (**Figure 6F**). On the other hand, *FTO* KD upregulated pathways involving Tumor Growth Factor beta, epithelial mesenchymal transition, KRAS signaling, estrogen response, UV response, myogenesis, and coagulation signaling (**Figure 6G**). These results indicate that FTO is involved in multiple signaling pathways; however, CS1 and *FTO* KD demonstrated commonalities of down-regulation of 4 key genes *EREG*, *KRAP*, *PDE4B* and *SLC38A2*.

## Discussion

FTO has been shown to play an important role in modulating fat mass, adipogenesis, and total body weight (39–41). Epidemiologic studies demonstrated that FTO single nucleotide polymorphisms (SNPs) are associated with the increased obesity and higher risk of multiple cancers including colorectal cancer (42). Specifically, there is a positive association between colorectal cancer and rs1558902, rs8050136, rs3751812, rs9939609 FTO SNPs in Japanese population (43) and the A allele of rs9939609 FTO SNP in Iranian population (44, 45). FTO, the first described demethylase of m6A mRNA, has been reported as an oncogene in different types of cancers. FTO is also found markedly upregulated in colorectal adenocarcinoma tissues (46). However, the role of FTO in colorectal cancer has not been fully investigated. In the present study, we show that FTO is expressed in various human colorectal cancer cell lines. We demonstrated that knockdown of *FTO* decreased cell proliferation, migration, invasion *in vitro* and suppressed tumor progression *in vivo*, which suggests that FTO may have an oncogenic role in colorectal cancer.

Since the 1980s, CS1 (or bisantrene) has shown some response in clinical trials as an anthracene compound for many types of cancer (47, 48). Recently, CS1 has been found to have high therapeutic efficacy against acute myeloid leukemia cells (32). In line with these findings, in our study, CS1 suppressed cell proliferation, induced cell cycle arrest in G2/M phase and promoted cell apoptosis in HCT116 cells *in vitro*. Moreover, CS1 treatment also inhibited tumor growth with minimal toxicity in colon cancer mouse models. 5-FU is the most common chemotherapeutic for CRC (49). However, the clinical efficacy is decreased in 5-FU resistant cells (50). Here, CS1 suppressed HCT116-5FUR cell viability in a dose-dependent manner (50-3200 nM, p<0.05 from 400 nM). These data suggested that CS1 might serve as a single alternative agent or in combination to overcome the resistance of 5-FU based therapies for CRC.

Using next-generation sequencing technology (NGS) with RNA-seq we investigated variations at the transcriptome level and differentially expressed genes (DEGs) of *FTO* KD in CRC. RNA-seq analysis of *FTO* KD revealed the distinct underlying molecular mechanisms and signaling pathways associated with antitumor activities in colorectal cancer cells through a set of differentially expressed genes including the top 4 down-regulated genes (*EREG*, *KRAP*, *PDE4B* and *SLC38A2*) confirmed by qPCR (**Figures 6D, E**). Epiregulin (EREG) is a ligand of epidermal growth factor receptor (EGFR), which is involved in RAS-RAF-MAPK and PI3K-AKT-mTOR signaling pathways regulating tumor proliferation, invasion, and migration (51). EREG is overexpressed in many types of cancer including colorectal cancer (52). Nearly 50% of colorectal cancers harbor *KRAS* mutations (53). CRC cells with mutant *KRAS* have been found to express higher autocrine levels of high-affinity EGFR ligands compared to wild-type *KRAS* (54). This strategy would be advantageous by targeting EREG in the 30% to 50% of CRC patients that harbor a *KRAS* mutation. KRAS-induced actin-interacting protein (KRAP), also named as actin-interacting protein sperm-specific antigen 2 (SSFA2), was originally identified as one of the genes that was up-regulated by activated KRAS in HCT116 cells (55). KRAP contributed to the regulation of filamentous actin and signals from the outside of the cells (56). Importantly, KRAP has been shown to be involved in cell proliferation in glioma (57) and oral squamous cell carcinoma (58). Those studies suggested that KRAP may serve as a potential target for colon cancer and other cancers. Our work demonstrates that FTO inhibition *via FTO* KD or *via* CS1 treatment leads to down-regulation of KRAP and may account for the mechanism of growth inhibition.

PDE4B belongs to the phosphodiesterase (PDE) family that catalyzes the hydrolysis of cyclic adenosine 3′,5’ monophosphate (cAMP) to AMP. cAMP is a ubiquitous second messenger and activated through its binding to activated protein kinase A (PKA), which is associated with various cellular processes including proliferation, differentiation, migration, and apoptosis (59). PDE4 is highly expressed in many kinds of cancer including colon cancer, melanoma, lymphoma, glioma, ovarian, brain tumors, and non-small cell lung cancer (60). Moreover, the expression of PDE4B is upregulated by oncogenic *KRAS* in HCT116 cells (61). PDE4B modulates the expression of MYC that leads to low intracellular cAMP levels, activates AKT/mTOR signaling, and promotes cell survival in colorectal cancer (62). These findings suggest that PDE4B may play a role in colon cancer and inhibition of PDE4B is a potential target for anticancer therapy We have demonstrated that *FTO* KD and inhibition with CS1 down-regulates PDE4B accounting for a possible mechanism of action of growth inhibition.

In order to promote proliferation and metastasis, tumor cells take up high levels of extracellular amino acids including glutamine (63). Glutamine is imported into cells *via* transporters, such as the Na+-coupled neutral amino acid transporters (SNATs) or the SLC38 superfamily (64). Among those transporters, SLC38A2 or SNAT2 have been reported to be overexpressed in tumors including prostate cancer (65), breast cancer (66), pancreatic cancer (67), and colorectal cancer (68). *KRAS* as one of the most prevalently mutated oncogenes in CRCs (69), has been found to upregulate glutamine transport, metabolism, and cell proliferation through mTOR activation leading to drug resistance (70, 71). Knockdown of amino acid transporters like SLC38A2, inhibit amino acid uptake and cell proliferation *via* mTOR suppression (68). This would be another effective strategy for colorectal cancer treatment. In this study we have demonstrated that FTO inhibition with CS1 or *FTO* KD leads to down regulation of SLC38A2.

There are multiple hallmarks of cancer during development of tumors including promoting cellular proliferation (72). MYC is a transcription factor regulating groups of genes (MYC targets) related to enhance cell growth in most types of human cancers (73). Consistently, our study revealed different signaling pathways downregulated by *FTO* KD, including MYC target V1 and MYC target V2. In CRCs, c−MYC has been shown to have a role in self−renewal, tumorigenicity, invasion and chemoresistance of cancer stem cells (74). In anti-EGFR targeted therapy, patients with high c-MYC expression had a markedly lower PFS, OS and more frequent metastases compared to patients with low c-MYC expression, which suggests a pivotal role of c-MYC in CRC resistance to EGFR inhibitors (75). Our RNA-seq data suggests that FTO inhibition leads to down regulation of the MYC pathway suggesting an additional potential mechanism of action.

In general, during malignant transformation, cancer cells undergo metabolic reprogramming to produce a huge amount of energy and biomass including a metabolic shift towards aerobic glycolysis characterized as the Warburg effect (76). On the other hand, oxidative phosphorylation (OXPHOS) is also a crucial pathway of cancer metabolism which supports progression and invasiveness in CRC (77, 78). OXPHOS has been found to be upregulated in CRC cells compared to healthy surrounding tissues, while the levels of glycolysis remained unchanged (79). A metabolic shift towards OXPHOS was linked to chemoresistance in CRC (77). Interestingly, *KRAS* mutations might be associated with an oxidative phenotype, while BRAF mutations with a glycolytic phenotype (80). In line with this, our study shows in our *KRAS* mutated cell line HCT116, *FTO* KD led to down regulation in oxidative phosphorylation pathways as seen by RNA-seq and no significant difference in glycolysis after treatment with the FTO inhibitor CS1 (Seahorse assay, data not shown). This data indicates that *FTO* KD impacts OXPHOS perhaps more than glycolysis.

Lastly, cell cycle regulation plays a key role in cell proliferation and in the progression of cancer *via* cell cycle-associated signaling pathways (81, 82). Our RNA-seq results indicated that the G2/M checkpoint was a downregulated pathway, in which CS1-treated HCT116 cells were induced into G2/M cell cycle arrest and subsequently the induction of cell apoptosis. Importantly, CS1 treatment suppressed the expression of CDC25C, one of the important regulators in the G2/M phase.

**Figure 7:**
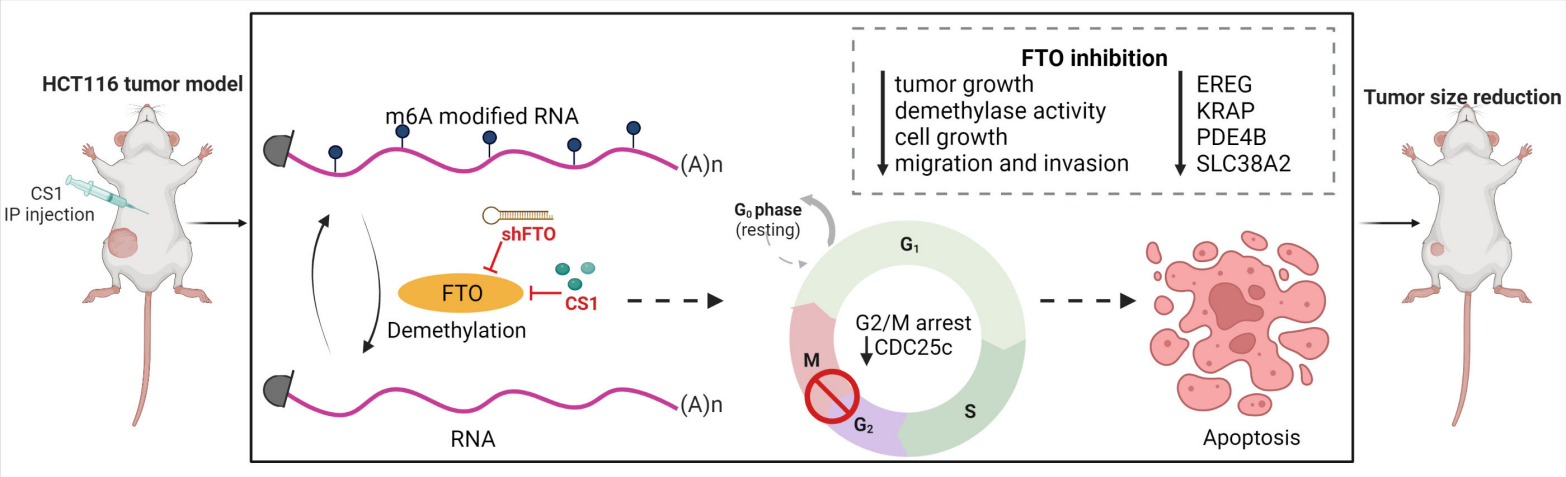
Targeting FTO in CRC treatment. FTO was identified as a potential oncogene in colorectal cancer cells and inhibition of FTO (by shFTO or CS1) significantly suppressed cancer cell proliferation, migration and invasion in vitro and tumor progression in vivo. FTO inhibition impacts multiple pathways related to EREG, KRAP, PDE4B and SLC38A2 that may account for its role as an oncogene. CS1, the FTO inhibitor, can be applied as a potential therapeutic agent for CRC treatment.

In summary, we identified FTO as a potential oncogene in colorectal cancer cells and we demonstrated that targeting FTO significantly suppressed cancer cell proliferation, migration and invasion *in vitro* and tumor progression *in vivo*. Additionally, we found that FTO inhibition impacts multiple pathways that may account for its role as an oncogene. Importantly, the FTO inhibitor, CS1 can be applied as a potential therapeutic agent for CRC treatment (**Figure 7**). Further work is needed to explore the roles of the most prominent downstream transcripts and signaling pathways that are impacted with changes in FTO expression and activity. This will lead to future clinical trials targeting FTO as a potential therapeutic strategy in the treatment of metastatic colorectal cancer.

## Data availability statement

The raw data supporting the conclusions of this article will be made available by the authors, without undue reservation.

## Ethics statement

The animal study was reviewed and approved by City of Hope IACUC protocol #16067.

## Author contributions

TP designed the experiments, performed experiments, analyzed the data, and prepared the manuscript. LM designed the experiments, provided supervision, prepared, and finalized the manuscript for submission. VN supported for the *in vivo* experiments. RS and JC provided the ideas and suggestions for FTO inhibitor in cancer. YL performed the m6A dot plot assay. YQ supported for the Seahorse assay. HQ, HC, and XW performed RNA-seq and data analysis. LJ, MF, DD and AG contributed to experimental design, data analysis and manuscript preparation. All the authors contributed to the article and approved the submitted version.

## References

[1] Siegel RL Miller KD Goding Sauer A Fedewa SA Butterly LF Anderson JC. Colorectal cancer statistics, 2020. CA Cancer J Clin (2020) 70(3):145–64. doi: 10.3322/caac.21601 32133645

[2] Siegel RL Miller KD Jemal A. Cancer statistics, 2020. CA Cancer J Clin (2020) 70(1):7–30. doi: 10.3322/caac.21590 31912902

[3] Yu T Guo F Yu Y Sun T Ma D Han J. Fusobacterium nucleatum promotes chemoresistance to colorectal cancer by modulating autophagy. Cell (2017) 170(3):548–63.e16. doi: 10.1016/j.cell.2017.07.008 28753429 PMC5767127

[4] Xie YH Chen YX Fang JY. Comprehensive review of targeted therapy for colorectal cancer. Signal transduction targeted Ther (2020) 5(1):22. doi: 10.1038/s41392-020-0116-z PMC7082344 32296018

[5] Boccaletto P Machnicka MA Purta E Piatkowski P Baginski B Wirecki TK. Modomics: A database of rna modification pathways. 2017 update. Nucleic Acids Res (2018) 46(D1):D303–D7. doi: 10.1093/nar/gkx1030 PMC5753262 29106616

[6] Frye M Harada BT Behm M He C. Rna modifications modulate gene expression during development. Science (2018) 361(6409):1346–9. doi: 10.1126/science.aau1646 PMC6436390 30262497

[7] Schumann U Shafik A Preiss T. Mettl3 gains R/W access to the epitranscriptome. Mol Cell (2016) 62(3):323–4. doi: 10.1016/j.molcel.2016.04.024 27153530

[8] Liu J Yue Y Han D Wang X Fu Y Zhang L. A Mettl3-Mettl14 complex mediates mammalian nuclear rna N6-adenosine methylation. Nat Chem Biol (2014) 10(2):93–5. doi: 10.1038/nchembio.1432 PMC3911877 24316715

[9] Ping X-L Sun B-F Wang L Xiao W Yang X Wang W-J. Mammalian wtap is a regulatory subunit of the rna N6-methyladenosine methyltransferase. Cell Res (2014) 24(2):177–89. doi: 10.1038/cr.2014.3 PMC3915904 24407421

[10] Jia G Fu Y Zhao X Dai Q Zheng G Yang Y. N6-methyladenosine in nuclear rna is a major substrate of the obesity-associated fto. Nat Chem Biol (2011) 7(12):885–7. doi: 10.1038/nchembio.687 PMC3218240 22002720

[11] Zheng G Dahl JA Niu Y Fedorcsak P Huang CM Li CJ. Alkbh5 is a mammalian rna demethylase that impacts rna metabolism and mouse fertility. Mol Cell (2013) 49(1):18–29. doi: 10.1016/j.molcel.2012.10.015 23177736 PMC3646334

[12] Wang X Lu Z Gomez A Hon GC Yue Y Han D. N6-Methyladenosine-Dependent regulation of messenger rna stability. Nature (2014) 505(7481):117–20. doi: 10.1038/nature12730 PMC3877715 24284625

[13] Alarcón CR Goodarzi H Lee H Liu X Tavazoie S Tavazoie SF. Hnrnpa2b1 is a mediator of M(6)a-dependent nuclear rna processing events. Cell (2015) 162(6):1299–308. doi: 10.1016/j.cell.2015.08.011 PMC4673968 26321680

[14] Huang H Weng H Sun W Qin X Shi H Wu H. Recognition of rna N(6)-methyladenosine by Igf2bp proteins enhances mrna stability and translation. Nat Cell Biol (2018) 20(3):285–95. doi: 10.1038/s41556-018-0045-z PMC5826585 29476152

[15] Luo J Yu J Peng X. Could partial nonstarch polysaccharides ameliorate cancer by altering M(6)a rna methylation in hosts through intestinal microbiota? Crit Rev Food Sci Nutr (2022) 62(30):8319–34. doi: 10.1080/10408398.2021.1927975 34036843

[16] Qiu FS He JQ Zhong YS Guo MY Yu CH. Implications of M6a methylation and microbiota interaction in non-small cell lung cancer: From basics to therapeutics. Front Cell infection Microbiol (2022) 12:972655. doi: 10.3389/fcimb.2022.972655 PMC9478539 36118041

[17] Chen S Zhang L Li M Zhang Y Sun M Wang L. Fusobacterium nucleatum reduces Mettl3-mediated M(6)a modification and contributes to colorectal cancer metastasis. Nat Commun (2022) 13(1):1248. doi: 10.1038/s41467-022-28913-5 35273176 PMC8913623

[18] Toh JDW Crossley SWM Bruemmer KJ Ge EJ He D Iovan DA. Distinct rna n-demethylation pathways catalyzed by nonheme iron Alkbh5 and fto enzymes enable regulation of formaldehyde release rates. Proc Natl Acad Sci USA (2020) 117(41):25284–92. doi: 10.1073/pnas.2007349117 PMC7568336 32989163

[19] Li Z Weng H Su R Weng X Zuo Z Li C. Fto plays an oncogenic role in acute myeloid leukemia as a N(6)-methyladenosine rna demethylase. Cancer Cell (2017) 31(1):127–41. doi: 10.1016/j.ccell.2016.11.017 PMC5234852 28017614

[20] Xu D Shao W Jiang Y Wang X Liu Y Liu X. Fto expression is associated with the occurrence of gastric cancer and prognosis. Oncol Rep (2017) 38(4):2285–92. doi: 10.3892/or.2017.5904 28849183

[21] Niu Y Lin Z Wan A Chen H Liang H Sun L. Rna N6-methyladenosine demethylase fto promotes breast tumor progression through inhibiting Bnip3. Mol Cancer (2019) 18(1):46. doi: 10.1186/s12943-019-1004-4 30922314 PMC6437932

[22] Yang S Wei J Cui Y-H Park G Shah P Deng Y. M6a mrna demethylase fto regulates melanoma tumorigenicity and response to anti-Pd-1 blockade. Nat Commun (2019) 10(1):2782. doi: 10.1038/s41467-019-10669-0 31239444 PMC6592937

[23] Zou D Dong L Li C Yin Z Rao S Zhou Q. The M(6)a eraser fto facilitates proliferation and migration of human cervical cancer cells. Cancer Cell Int (2019) 19:321. doi: 10.1186/s12935-019-1045-1 31827395 PMC6888952

[24] Zhuang C Zhuang C Luo X Huang X Yao L Li J. N6-methyladenosine demethylase fto suppresses clear cell renal cell carcinoma through a novel fto-Pgc-1α signalling axis. J Cell Mol Med (2019) 23(3):2163–73. doi: 10.1111/jcmm.14128 PMC6378205 30648791

[25] Rong ZX Li Z He JJ Liu LY Ren XX Gao J. Downregulation of fat mass and obesity associated (Fto) promotes the progression of intrahepatic cholangiocarcinoma. Front Oncol (2019) 9:369. doi: 10.3389/fonc.2019.00369 31143705 PMC6521779

[26] Liu X Liu J Xiao W Zeng Q Bo H Zhu Y. Sirt1 regulates N(6) -methyladenosine rna modification in hepatocarcinogenesis by inducing Ranbp2-dependent fto sumoylation. Hepatol (Baltimore Md) (2020) 72(6):2029–50. doi: 10.1002/hep.31222 32154934

[27] Huang H Wang Y Kandpal M Zhao G Cardenas H Ji Y. (6)-methyladenosine modifications inhibit ovarian cancer stem cell self-renewal by blocking camp signaling. Cancer Res (2020) 80(16):3200–14. doi: 10.1158/0008-5472.Can-19-4044 PMC7442742 32606006

[28] Lin Z Wan AH Sun L Liang H Niu Y Deng Y. N6-methyladenosine demethylase FTO enhances chemo-resistance in colorectal cancer through SIVA1-mediated apoptosis. Mol Ther (2023) 31(2):517–34. doi: 10.1016/j.ymthe.2022.10.012 PMC9931553 36307991

[29] Wang J Qiao Y Sun M Sun H Xie F Chang H. Fto promotes colorectal cancer progression and chemotherapy resistance *Via* demethylating G6pd/Parp1. Clin Trans Med (2022) 12(3):e772. doi: 10.1002/ctm2.772 PMC8926902 35297218

[30] Ruan DY Li T Wang YN Meng Q Li Y Yu K. Fto downregulation mediated by hypoxia facilitates colorectal cancer metastasis. Oncogene (2021) 40(33):5168–81. doi: 10.1038/s41388-021-01916-0 PMC8376648 34218271

[31] Relier S Ripoll J Guillorit H Amalric A Achour C Boissière F. Fto-mediated cytoplasmic M(6)a(M) demethylation adjusts stem-like properties in colorectal cancer cell. Nat Commun (2021) 12(1):1716. doi: 10.1038/s41467-021-21758-4 33741917 PMC7979729

[32] Su R Dong L Li Y Gao M Han L Wunderlich M. Targeting fto suppresses cancer stem cell maintenance and immune evasion. Cancer Cell (2020) 38(1):79–96.e11. doi: 10.1016/j.ccell.2020.04.017 32531268 PMC7363590

[33] Martin M. Cutadapt removes adapter sequences from high-throughput sequencing reads. EMBnet J (2011) 17(1):10–2. doi: 10.14806/ej.17.1.200

[34] Dobin A Davis CA Schlesinger F Drenkow J Zaleski C Jha S. Star: Ultrafast universal rna-seq aligner. Bioinformatics (2013) 29(1):15–21. doi: 10.1093/bioinformatics/bts635 23104886 PMC3530905

[35] Li B Dewey CN. Rsem: Accurate transcript quantification from rna-seq data with or without a reference genome. BMC Bioinf (2011) 12:323. doi: 10.1186/1471-2105-12-323 PMC3163565 21816040

[36] Thorvaldsdóttir H Robinson JT Mesirov JP. Integrative genomics viewer (Igv): High-performance genomics data visualization and exploration. Brief Bioinform (2013) 14(2):178–92. doi: 10.1093/bib/bbs017 PMC3603213 22517427

[37] Subramanian A Tamayo P Mootha VK Mukherjee S Ebert BL Gillette MA. Gene set enrichment analysis: A knowledge-based approach for interpreting genome-wide expression profiles. Proc Natl Acad Sci USA (2005) 102(43):15545–50. doi: 10.1073/pnas.0506580102 PMC1239896 16199517

[38] DiPaola RS. To arrest or not to G2-m cell-cycle arrest. Clin Cancer Res (2002) 8(11):3311. 12429616

[39] Fischer J Koch L Emmerling C Vierkotten J Peters T Brüning JC. Inactivation of the fto gene protects from obesity. Nature (2009) 458(7240):894–8. doi: 10.1038/nature07848 19234441

[40] McMurray F Church CD Larder R Nicholson G Wells S Teboul L. Adult onset global loss of the fto gene alters body composition and metabolism in the mouse. PloS Genet (2013) 9(1):e1003166. doi: 10.1371/journal.pgen.1003166 23300482 PMC3536712

[41] Merkestein M Laber S McMurray F Andrew D Sachse G Sanderson J. Fto influences adipogenesis by regulating mitotic clonal expansion. Nat Commun (2015) 6(1):6792. doi: 10.1038/ncomms7792 25881961 PMC4410642

[42] Deng X Su R Stanford S Chen J. Critical enzymatic functions of fto in obesity and cancer. Front Endocrinol (Lausanne) (2018) 9:396. doi: 10.3389/fendo.2018.00396 30105001 PMC6077364

[43] Yamaji T Iwasaki M Sawada N Shimazu T Inoue M Tsugane S. Fat mass and obesity-associated gene polymorphisms, pre-diagnostic plasma adipokine levels and the risk of colorectal cancer: The Japan public health center-based prospective study. PloS One (2020) 15(2):e0229005. doi: 10.1371/journal.pone.0229005 32053666 PMC7017986

[44] Gholamalizadeh M Akbari ME Doaei S Davoodi SH Bahar B Tabesh GA. The association of fat-mass-and obesity-associated gene polymorphism (Rs9939609) with colorectal cancer: A case-control study. Front Oncol (2021) 11:732515. doi: 10.3389/fonc.2021.732515 34650918 PMC8506030

[45] Gholamalizadeh M Tabrizi R Bourbour F Rezaei S Pourtaheri A Badeli M. Are the fto gene polymorphisms associated with colorectal cancer? a meta-analysis. J Gastrointest Cancer (2021) 52(3):846–53. doi: 10.1007/s12029-021-00651-9 34212310

[46] Shen XP Ling X Lu H Zhou CX Zhang JK Yu Q. Low expression of microrna-1266 promotes colorectal cancer progression *Via* targeting fto. Eur Rev Med Pharmacol Sci (2018) 22(23):8220–6. doi: 10.26355/eurrev_201812_16516 30556861

[47] Cowan JD Gehan E Rivkin SE Jones SE. Phase ii trial of bisantrene in patients with advanced sarcoma: A southwest oncology group study. Cancer Treat Rep (1986) 70(5):685–6. 3708622

[48] Miller TP Cowan JD Neilan BA Jones SE. A phase ii study of bisantrene in malignant lymphomas. a southwest oncology group study. Cancer Chemother Pharmacol (1986) 16(1):67–9. doi: 10.1007/bf00255289 3940222

[49] Longley DB Harkin DP Johnston PG. 5-fluorouracil: Mechanisms of action and clinical strategies. Nat Rev Cancer (2003) 3(5):330–8. doi: 10.1038/nrc1074 12724731

[50] Blondy S David V Verdier M Mathonnet M Perraud A Christou N. 5-fluorouracil resistance mechanisms in colorectal cancer: From classical pathways to promising processes. Cancer Sci (2020) 111(9):3142–54. doi: 10.1111/cas.14532 PMC7469786 32536012

[51] Zhao B Wang L Qiu H Zhang M Sun L Peng P. Mechanisms of resistance to anti-egfr therapy in colorectal cancer. Oncotarget (2017) 8(3):3980–4000. doi: 10.18632/oncotarget.14012 28002810 PMC5354808

[52] Kuramochi H Nakajima G Kaneko Y Nakamura A Inoue Y Yamamoto M. Amphiregulin and epiregulin mrna expression in primary colorectal cancer and corresponding liver metastases. BMC Cancer (2012) 12(1):88. doi: 10.1186/1471-2407-12-88 22409860 PMC3317853

[53] Xie J Xia L Xiang W He W Yin H Wang F. Metformin selectively inhibits metastatic colorectal cancer with the kras mutation by intracellular accumulation through silencing Mate1. Proc Natl Acad Sci USA (2020) 117(23):13012. doi: 10.1073/pnas.1918845117 32444490 PMC7293710

[54] Lee HW Son E Lee K Lee Y Kim Y Lee JC. Promising therapeutic efficacy of Gc1118, an anti-egfr antibody, against kras mutation-driven colorectal cancer patient-derived xenografts. Int J Mol Sci (2019) 20(23):5894. doi: 10.3390/ijms20235894 31771279 PMC6928876

[55] Inokuchi J Komiya M Baba I Naito S Sasazuki T Shirasawa S. Deregulated expression of krap, a novel gene encoding actin-interacting protein, in human colon cancer cells. J Hum Genet (2004) 49(1):46–52. doi: 10.1007/s10038-003-0106-3 14673706

[56] Fujimoto T Koyanagi M Baba I Nakabayashi K Kato N Sasazuki T. Analysis of krap expression and localization, and genes regulated by krap in a human colon cancer cell line. J Hum Genet (2007) 52(12):978–84. doi: 10.1007/s10038-007-0204-8 17934691

[57] Zhu A Li X Wu H Miao Z Yuan F Zhang F. Molecular mechanism of Ssfa2 deletion inhibiting cell proliferation and promoting cell apoptosis in glioma. Pathol Res Pract (2019) 215(3):600–6. doi: 10.1016/j.prp.2018.12.035 30712887

[58] Zhu L Zhang L Tang Y Zhang F Wan C Xu L. Microrna−363−3p inhibits tumor cell proliferation and invasion in oral squamous cell carcinoma cell lines by targeting Ssfa2. Exp Ther Med (2021) 21(6):549. doi: 10.3892/etm.2021.9981 33850521 PMC8027732

[59] Chin KV Yang WL Ravatn R Kita T Reitman E Vettori D. Reinventing the wheel of cyclic amp: Novel mechanisms of camp signaling. Ann N Y Acad Sci (2002) 968:49–64. doi: 10.1111/j.1749-6632.2002.tb04326.x 12119267

[60] Mehta A Patel BM. Therapeutic opportunities in colon cancer: Focus on phosphodiesterase inhibitors. Life Sci (2019) 230:150–61. doi: 10.1016/j.lfs.2019.05.043 31125564

[61] Tsunoda T Ota T Fujimoto T Doi K Tanaka Y Yoshida Y. Inhibition of phosphodiesterase-4 (Pde4) activity triggers luminal apoptosis and akt dephosphorylation in a 3-d colonic-crypt model. Mol Cancer (2012) 11:46. doi: 10.1186/1476-4598-11-46 22830422 PMC3439292

[62] Kim DU Kwak B Kim SW. Phosphodiesterase 4b is an effective therapeutic target in colorectal cancer. Biochem Biophys Res Commun (2019) 508(3):825–31. doi: 10.1016/j.bbrc.2018.12.004 30528730

[63] Rubin H. Deprivation of glutamine in cell culture reveals its potential for treating cancer. Proc Natl Acad Sci USA (2019) 116(14):6964–8. doi: 10.1073/pnas.1815968116 PMC6452721 30877243

[64] Kandasamy P Gyimesi G Kanai Y Hediger MA. Amino acid transporters revisited: New views in health and disease. Trends Biochem Sci (2018) 43(10):752–89. doi: 10.1016/j.tibs.2018.05.003 30177408

[65] Okudaira H Shikano N Nishii R Miyagi T Yoshimoto M Kobayashi M. Putative transport mechanism and intracellular fate of trans-1-Amino-3-18f-Fluorocyclobutanecarboxylic acid in human prostate cancer. J Nucl Med (2011) 52(5):822–9. doi: 10.2967/jnumed.110.086074 21536930

[66] Morotti M Bridges E Valli A Choudhry H Sheldon H Wigfield S. Hypoxia-induced switch in Snat2/Slc38a2 regulation generates endocrine resistance in breast cancer. Proc Natl Acad Sci USA (2019) 116(25):12452–61. doi: 10.1073/pnas.1818521116 PMC6589752 31152137

[67] Parker SJ Amendola CR Hollinshead KER Yu Q Yamamoto K Encarnación-Rosado J. Selective alanine transporter utilization creates a targetable metabolic niche in pancreatic cancer. Cancer Discov (2020) 10(7):1018–37. doi: 10.1158/2159-8290.CD-19-0959 PMC7334074 32341021

[68] Kandasamy P Zlobec I Nydegger DT Pujol-Giménez J Bhardwaj R Shirasawa S. Oncogenic kras mutations enhance amino acid uptake by colorectal cancer cells *Via* the hippo signaling effector Yap1. Mol Oncol (2021) 15(10):2782–800. doi: 10.1002/1878-0261.12999 PMC8486573 34003553

[69] Knickelbein K Zhang L. Mutant kras as a critical determinant of the therapeutic response of colorectal cancer. Genes Dis (2015) 2(1):4–12. doi: 10.1016/j.gendis.2014.10.002 25815366 PMC4372129

[70] Yoo HC Yu YC Sung Y Han JM. Glutamine reliance in cell metabolism. Exp Mol Med (2020) 52(9):1496–516. doi: 10.1038/s12276-020-00504-8 PMC8080614 32943735

[71] Scalise M Pochini L Galluccio M Console L Indiveri C. Glutamine transporters as pharmacological targets: From function to drug design. Asian J Pharm Sci (2020) 15(2):207–19. doi: 10.1016/j.ajps.2020.02.005 PMC7193454 32373200

[72] Hanahan D Weinberg Robert A. Hallmarks of cancer: The next generation. Cell (2011) 144(5):646–74. doi: 10.1016/j.cell.2011.02.013 21376230

[73] Dang CV O’Donnell KA Zeller KI Nguyen T Osthus RC Li F. The c-myc target gene network. Semin Cancer Biol (2006) 16(4):253–64. doi: 10.1016/j.semcancer.2006.07.014 16904903

[74] Zhang HL Wang P Lu MZ Zhang SD Zheng L. C−Myc maintains the Self−Renewal and chemoresistance properties of colon cancer stem cells. Oncol Lett (2019) 17(5):4487–93. doi: 10.3892/ol.2019.10081 PMC6444394 30944638

[75] Strippoli A Cocomazzi A Basso M Cenci T Ricci R Pierconti F. C-myc expression is a possible keystone in the colorectal cancer resistance to egfr inhibitors. Cancers (2020) 12(3):638. doi: 10.3390/cancers12030638 32164324 PMC7139615

[76] Koppenol WH Bounds PL Dang CV. Otto Warburg’s contributions to current concepts of cancer metabolism. Nat Rev Cancer (2011) 11(5):325–37. doi: 10.1038/nrc3038 21508971

[77] Denise C Paoli P Calvani M Taddei ML Giannoni E Kopetz S. 5-fluorouracil resistant colon cancer cells are addicted to oxphos to survive and enhance stem-like traits. Oncotarget (2015) 6(39):41706–21. doi: 10.18632/oncotarget.5991 PMC4747183 26527315

[78] Lin CS Liu LT Ou LH Pan SC Lin CI Wei YH. Role of mitochondrial function in the invasiveness of human colon cancer cells. Oncol Rep (2018) 39(1):316–30. doi: 10.3892/or.2017.6087 29138850

[79] Chekulayev V Mado K Shevchuk I Koit A Kaldma A Klepinin A. Metabolic remodeling in human colorectal cancer and surrounding tissues: Alterations in regulation of mitochondrial respiration and metabolic fluxes. Biochem Biophys Rep (2015) 4:111–25. doi: 10.1016/j.bbrep.2015.08.020 PMC5668899 29124194

[80] Rebane-Klemm E Truu L Reinsalu L Puurand M Shevchuk I Chekulayev V. Mitochondrial respiration in kras and braf mutated colorectal tumors and polyps. Cancers (2020) 12(4):815. doi: 10.3390/cancers12040815 32231083 PMC7226330

[81] Williams GH Stoeber K. The cell cycle and cancer. J Pathol (2012) 226(2):352–64. doi: 10.1002/path.3022 21990031

[82] Otto T Sicinski P. Cell cycle proteins as promising targets in cancer therapy. Nat Rev Cancer (2017) 17(2):93–115. doi: 10.1038/nrc.2016.138 28127048 PMC5345933

